# A Review of the Segmental Diameter of the Healthy Human Spinal Cord

**DOI:** 10.3389/fneur.2016.00238

**Published:** 2016-12-23

**Authors:** Arvid Frostell, Ramil Hakim, Eric Peter Thelin, Per Mattsson, Mikael Svensson

**Affiliations:** ^1^Department of Clinical Neuroscience, Karolinska Institutet, Stockholm, Sweden; ^2^Department of Neurosurgery, Karolinska University Hospital, Stockholm, Sweden

**Keywords:** spinal cord, reference point conversion, morphometry, segmental diameter, vertebral segment, neuronal segment

## Abstract

Knowledge of the average size and variability of the human spinal cord can be of importance when treating pathological conditions in the spinal cord. Data on healthy human spinal cord morphometrics have been published for more than a century using different techniques of measurements, but unfortunately, comparison of results from different studies is difficult because of the different anatomical landmarks used as reference points along the craniocaudal axis for the measurements. The aim of this review was to compute population estimates of the transverse and anteroposterior diameter of the human spinal cord by comparing and combining previously published data on a normalized craniocaudal axis. We included 11 studies presenting measurements of spinal cord cross-sectional diameters, with a combined sample size ranging from 15 to 488 subjects, depending on spinal cord level. Based on five published studies presenting data on the lengths of the segments of the spinal cord and vertebral column, we calculated the relative positions of all spinal cord neuronal segments and vertebral bony segments and mapped measurements of spinal cord size to a normalized craniocaudal axis. This mapping resulted in better alignment between studies and allowed the calculation of weighted averages and standard deviations (SDs) along the spinal cord. These weighted averages were smoothed using a generalized additive model to yield continuous population estimates for transverse and anteroposterior diameter and associated SDs. The spinal cord had the largest transverse diameter at spinal cord neuronal segment C5 (13.3 ± 2.2), decreased to segment T8 (8.3 ± 2.1), and increased slightly again to 9.4 ± 1.5 at L3. The anteroposterior diameter showed less variation in size along the spinal cord at C5 (7.4 ± 1.6), T8 (6.3 ± 2.0), and L3 (7.5 ± 1.6). All estimates are presented in millimeters ± 2 SDs. We conclude that segmental transverse and anteroposterior diameters of the healthy human spinal cord from different published sources can be combined on a normalized craniocaudal axis and yield meaningful population estimates. These estimates could be useful in routine management of patients with neurodegenerative diseases as well as for clinical research and experimental applications aimed at surgical spinal cord repair.

## Introduction

The spinal cord constitutes the main channel of afferent and efferent signaling between the body and the brain, and pathology in the spinal cord typically leads to significant lifelong functional deficits in afflicted patients, regardless of traumatic or autoimmune etiology (e.g., spinal cord injury and multiple sclerosis).

Knowledge of the average size and variation of the human spinal cord can be of importance when treating pathological conditions in the spinal cord. It is known that patients suffering from multiple sclerosis have a reduced cross-sectional area compared to healthy matched controls (1). These case-control studies typically suffer from low power and without population estimates, it can be difficult to determine whether a specific patient should be considered to have a pathologically small spinal cord. Furthermore, many experimental strategies for the treatment of acute and chronic traumatic spinal cord injuries are in different phases of development (2). In all studies where a premade device, instrumentation, or otherwise physical object needs to be applied to the spinal cord, the population estimates of spinal cord size are of importance because they represent the variation in physical dimensions that will be encountered when operating on patients.

Data on healthy human spinal cord morphometrics have been published for more than a century using different techniques of measurements and different reference points along the craniocaudal axis of the spinal cord. Imaging techniques such as computed tomography (CT) can be used to detect soft-tissue changes and damage to vertebrae, while magnetic resonance imaging (MRI) is most appropriate for defining neuronal tissue (3–5). Voxel-based techniques, implemented on MRI images, are available for spinal cord cross-sectional area measurement as a means for fast and comprehensive assessment of volumetric changes (6). Although these imaging techniques give important information about the existence of damage to vertebrae and/or neuronal tissue, the techniques do not provide an exact methodology for determining the location and morphometries of an affected spinal cord neuronal segment. The main disadvantage with the radiological approaches is inadequate resolution. Therefore, histological studies have also been implemented. However, neuronal tissue does not retain shape postmortem, introducing other technical challenges and possible bias.

The human spinal cord is made up of 30 neuronal segments distributed along the spinal cord in eight cervical, 12 thoracic, 5 lumbar, and 5 sacral segments. The spinal cord is positioned in the vertebral canal of the vertebral column. The vertebral column is made up of 24 segments with 7 cervical, 12 thoracic, and 7 lumbar segments. However, the spinal cord terminates approximately between lumbar vertebrae L1 and L2, and, therefore, the 30 spinal cord neuronal segments are distributed over 20 vertebral bony segments. Most radiological techniques cannot determine spinal cord neuronal segment level, but instead, rely on reporting the vertebral bony segment level. In contrast, postmortem studies commonly rely on the spinal rootlets for determining spinal cord neuronal segmental level. Comparison between and combination of results from different studies are inherently difficult because of the diverse anatomical landmarks used for the measurements.

This review sought to compute population estimates of the transverse and anteroposterior diameter of the entire human spinal cord by comparing and combining previously published data on a normalized craniocaudal axis.

## Materials and Methods

### Studies and Data

#### Inclusion in the Analysis

We searched PubMed for original research publications reporting morphometric data on the human spinal cord. Studies not found in PubMed but referred to in the included studies were also added. Table 1 shows the studies presenting cross-sectional measurements of the human spinal cord, while Table 2 shows the studies presenting longitudinal measurements along the craniocaudal axis of the spinal cord neuronal segments. We also included three studies presenting the length of the vertebral bony segments in Table 2 (7–9).

**Table 1:** **Studies presenting measurements of the cross-sectional diameter of the human spinal cord included in this review**.

Article	Method	Reference point	Segments measured	Number of subjects
Elliot (23)	Postmortem examination	Neuronal	C5, T6, L5	102
Nordqvist (16)	Postmortem X-ray myelography	Vertebral	C2-L1	18
Thijssen et al. (17)	*In vivo* CT myelography	Vertebral	C1-T1	20
Lamont et al. (18)	*In vivo* X-ray myelography	Vertebral	C1-T1	69
Sherman et al. (4)	*In vivo* MRI	Vertebral	C1-T3	66
Kameyama et al. (19)	Postmortem examination	Neuronal	C2-T1	14
Kameyama et al. (19)	Postmortem examination	Neuronal	C7	152
Kameyama et al. (20)	Postmortem examination	Neuronal	C2-S3	12
Fountas et al. (21)	*In vivo* CT myelography	Vertebral	C2-C7	102
Ko et al. (22)	Postmortem examination	Neuronal	C3-S5	15
Zaaroor et al. (5)	*In vivo* MRI	Vertebral	C1-L1	20

*C, cervical; T, thoracic; L, lumbar; CT, computed tomography; MRI, magnetic resonance imaging*.

**Table 2:** **Studies presenting measurements of the length of the human spinal cord neuronal segments and verebral bony segments included in this review**.

Article	Method	Reference point	Segments measured	Number of subjects
Donaldson and Davis (24)	Postmortem examination	Neuronal	C1-S5	4
Panjabi et al. (7–9)	Postmortem examination	Vertebral	C2-L5	12
Ko et al. (22)	Postmortem examination	Neuronal	C3-S5	15
Cadotte et al. (10)	*In vivo* MRI	Neuronal/vertebral	C3-C8/C3-C7	10

*C, cervical; L, lumbar; S, sacral*.

#### Extracting Data from Studies

Most of the studies included did not present the raw data from their measurements; instead, averages and standard deviations (SDs) were provided. Some studies presented their data in graphical instead of numerical format. To ensure correct extraction of data from these studies, we imported images of the graphs into a CAD-program (Rhino 5 for Mac, Robert McNeel & Associates) and used the internal measurements tool to extract the precise values from the graphs.

### Relative Lengths of Segments

#### Calculating Relative Lengths of Spinal Cord Neuronal Segments

Using the data from the studies in Table 2, we calculated the relative length of each spinal cord neuronal segment by simply dividing the length of each segment by the total length of the spinal cord. By estimating the segmental diameter, the measurements from the different studies were weighted according to the number of subjects (i.e., individuals) in each study.

#### Calculating Relative Lengths of Vertebral Bony Segments and Aligning Spinal Cord Neuronal Segments

Using the data from the studies in Table 2, we also calculated the relative length of each vertebral bony segment using the same method described above for the neuronal segments. A vertebral bony segment was defined as the vertebra and half of the two adjacent intervertebral disks. The disks were assumed to increase in size proportionally to the vertebrae.

There were no measurements for vertebral segments C1 and C2 in the studies that we found. Their respective ratios were approximated by aligning vertebral bony segments with spinal cord neuronal segments in the cervical region according to Cadotte et al. (10). Specifically, the distance between the midpoint of spinal cord neuronal segment C3 and vertebral bony segment C3 was set to 1.3 times the distance between spinal cord neuronal segments C3 and C4. Finally, we assumed that both the spinal cord and the vertebral column terminated at the same cranial level and divided the distance equally between C1 and C2 vertebral bony segments. Therefore, our calculated relative sizes of C1 and C2 should be considered approximations and interpreted with care.

### Relative Positioning of Segments

#### Relative Positioning and Scaling of Spinal Cord Neuronal Segments and Vertebral Bony Segments

To align the spinal cord neuronal segments with the vertebral bony segments, we multiplied all cumulative percentages for vertebral bony segments by 1.29. This scaling factor was calculated by dividing the cumulative percentage of entire spinal cord (100%) with the cumulative percentage of the vertebral column at vertebral bony segment L1. This new scaling of vertebral bony segments set the caudal end of the L1 vertebral bony segment equal to the caudal end of spinal cord neuronal segment S5. As a result, the positioning depends on knowledge of the positions of the C3 and C4 spinal cord neuronal segments relative to the C3 vertebral bony segment presented by Cadotte et al. (10) and the level of termination of the spinal cord between vertebral bony segments L1 and L2 (11).

#### Corrected Positioning of Transverse Diameter Measurements of the Human Cervical Spinal Cord

**Figure 1:**
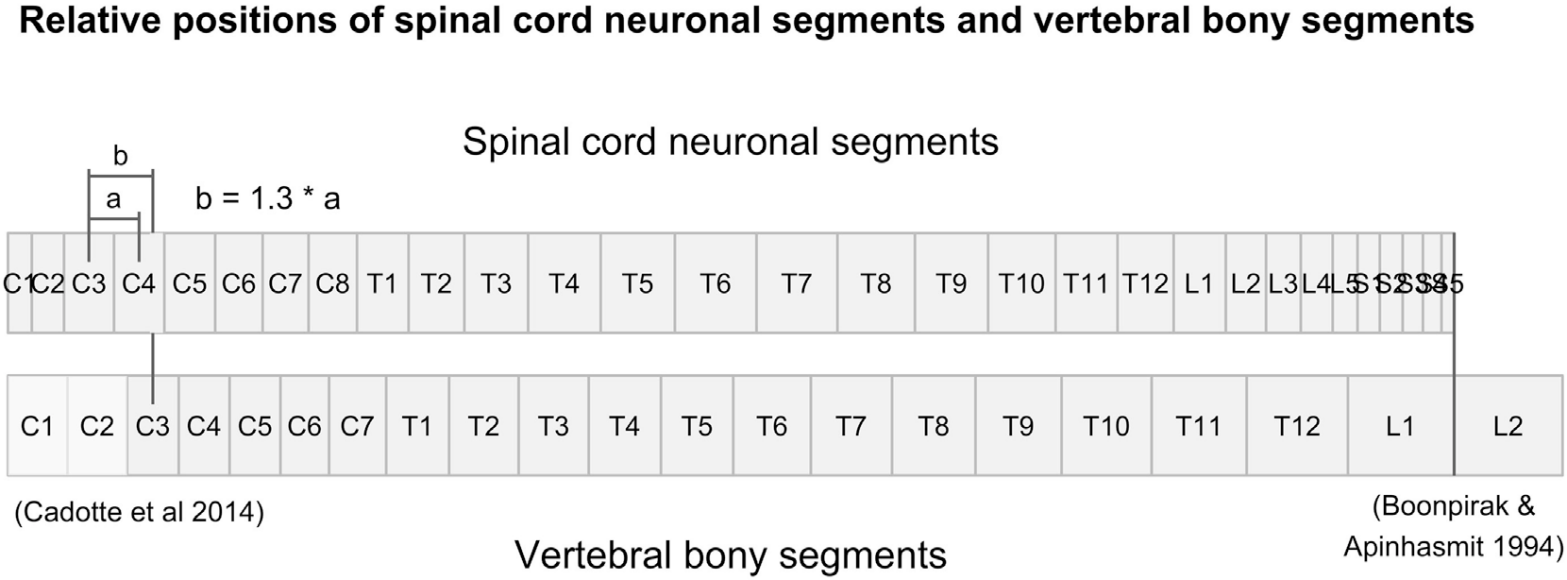
**Figure illustrates the relative positions of each neuronal spinal cord segment and vertebral bony segment in the human spine**. Relative positions were calculated using data from Tables 3 and 4, together with relative positions of the C3-vertebral and C3-neuronal segment (10) and the mean spinal cord termination in the spinal canal at L1/L2 (11).

The positions of the segments shown in Figure 1 were used to find the correct relative positions of each cross-sectional measurement along a normalized craniocaudal axis of the human spinal cord. Each measurement was placed as closely as possible to the anatomical position described by the original authors, with respect to the type of segmental reference used in the study (spinal cord neuronal segment or vertebral bony segment) as well as the positioning on that specific segment (cranial end of segment, midpoint of segment, or caudal end of segment).

#### Evaluating the Corrected Positioning of Measurements

**Figure 2:**
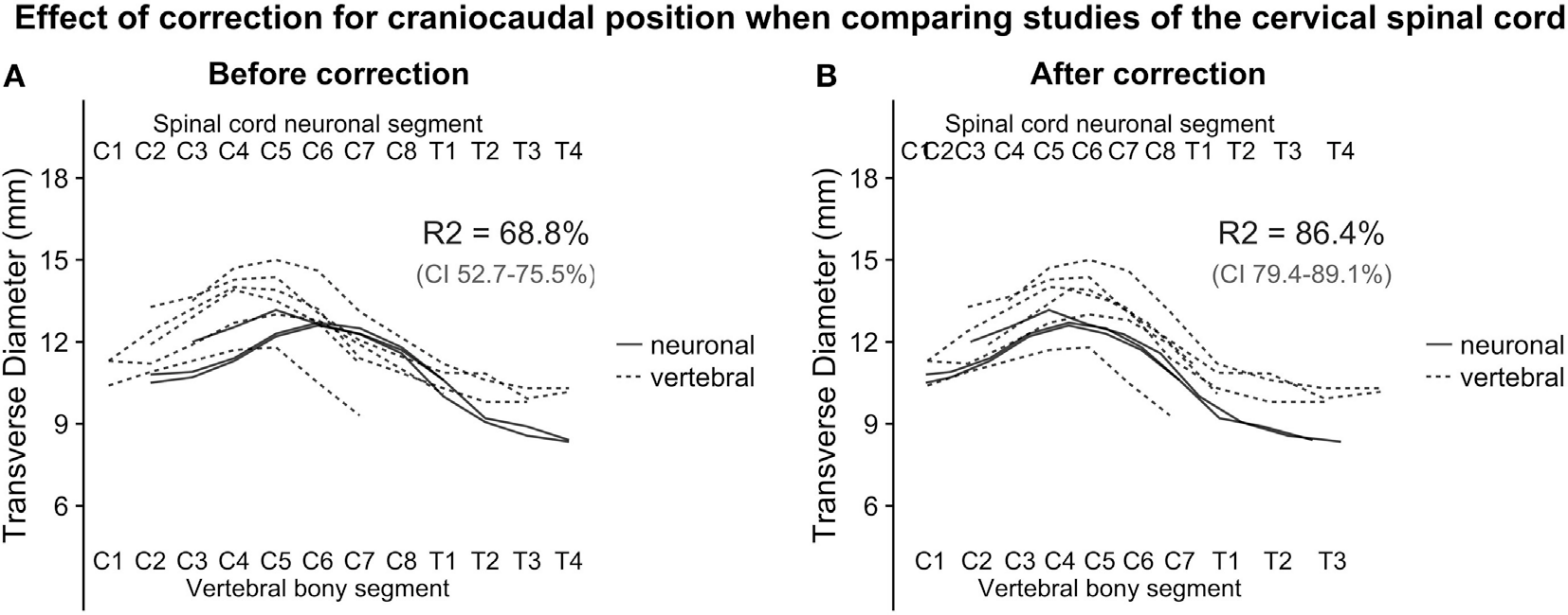
**(A,B)**: with the relative positions of spinal cord neuronal segments and vertebral bony segments illustrated in Figure 1, we plotted measurements of the transverse diameter of the cervical spinal cord. In panel **(A)**, the measurement of the transverse diameter of the spinal cord is not corrected for craniocaudal position, and, therefore, the cervical intumescence is misaligned between studies with different reference points. In panel **(B)** (corrected for craniocaudal position), the intumescence appears aligned. The difference in alignment was tested by fitting a second degree polynomial regression to the data points. Bootstrapping confidence intervals for the two estimates of R2 (for corrected and uncorrected, respectively) showed that the confidence intervals were non-overlapping, indicating a substantial improvement of alignment after correction.

To estimate the effect of adjusted craniocaudal positions on transverse diameter measurements of the human cervical spinal cord shown in Figure 2, we fitted a linear regression model, before and after correction of craniocaudal position:

$$\begin{array}{l} Transverse\;Diameter\sim \beta_{1}*Position\;+\;\beta_{2}*Position^{2}+\beta_{3}*Study \end{array}$$

The squared term was added because the cervical spinal cord transverse diameter approximates the shape of a second-degree polynomial, and the dummy term study was added to correct for differences in intercept between the studies. Adjusted *R*-squared was used as a measure of alignment of the cervical intumescences between studies. Confidence intervals for the adjusted *R*-squared were estimated using a 1,000 iteration bootstrap.

### Weighted Averages

#### Calculating Weighted Averages and Variances along the Spinal Cord

To combine the cross-sectional measurements of the human spinal cord from all studies into single estimates, we calculated a moving weighted average. First, measurements from all studies were aligned along their correct position on our corrected craniocaudal axis described above and in Figures 1 and 2. Thereafter, starting at the cranial end, four consecutive measurements of spinal cord diameter were combined into a single average, weighted by the number of subjects in the comprising studies for the four included measurements. The average position along the craniocaudal axis of the four measurements was used as the new position for the weighted average. Next, the most cranial of the four measurements was dropped, and the closest measurement caudal to the three remaining measurements was included to create a new group of four measurements, with a new weighted average and a new position along the craniocaudal axis.

Moving weighted variances were calculated using the same method as described for the moving weighted averages. The calculated variances were then converted to weighted SDs.

### Population Estimates

#### Constructing Continuous Population Estimates with a Generalized Additive Model

To construct continuous population estimates and achieve further smoothing, a generalized additive model was used to fit the weighted averages and weighted SDs. We used the smoothing function of ggplot2 (12) in *R* (13) with the formula *y* ~ *s*(*x, k* = 12), allowing a 12° polynomial function to fit the data.

#### Extracting Point Values of Continuous Population Estimates along the Spinal Cord

To facilitate comparison between our continuous population estimates and other studies, we extracted values for each spinal cord neuronal segment and vertebral bony segment. The number of subjects measured for a given segment was defined as the total number of subjects included in any study with a calculated craniocaudal position inside the cranial and caudal limits of the segment in question. This was used as an approximation of sample size, as there is no obvious way of calculating exact sample size for different portions of a smoothing function.

#### Number of Measurements and Relative Contribution of Studies along the Spinal Cord

To present the total number of measurements included at different points along the craniocaudal axis of the spinal cord, we plotted the total number of measurements in each vertebral bony segment in Figure 6A. Figure 6B shows the relative contribution of each included study along the spinal cord, and Figure 6C shows the relative contribution of different methods for obtaining measurements.

### Software

Data was gathered in Microsoft Excel and stored as comma-separated values (.csv), all calculations were performed in *R* (13), and graphs were produced with the ggplot2 and cowplot packages (12, 14). Bootstrapping was performed with the boot package (15).

## Results

### Studies and Data

#### Studies Included in the Analysis

Data on the diameter of the healthy human spinal cord were available from various published sources, covering more than 100 years of research and various acquisition methodologies (4, 5, 16–24). The published papers differed in terms of methodology of measurement, anatomical reference points, segments measured, and the number of subjects included. Six published papers reported data on the lengths of the spinal cord neuronal and vertebral bony segments (7–9, 22).

All studies reported SDs of measurements, except for the following: Fountas et al. (21), Ko et al. (22), and Nordqvist (16). Donaldson and Davis (24) did not report SDs, but all raw data was presented in the paper, so the SDs could be computed. Raw data for measurements of anteroposterior diameter in Nordqvist (16) was also presented in the paper, but not for transverse diameter. Tables 1 and 2 give an overview of included studies.

### Relative Lengths of Segments

#### Relative Length of Spinal Cord Neuronal Segments

The longest spinal cord neuronal segments were found in the thoracic spinal cord, and each segment constituted approximately 5% of the whole spinal cord. Multiplying the relative length of a spinal cord neuronal segment in Table 3 with the average length of the spinal cord yielded segments lengths well above 2 cm in the thoracic spinal cord and around 1.5 cm in the cervical spinal cord. The calculated relative lengths of each spinal cord neuronal segment are presented in Table 3.

**Table 3:** **Relative lengths of human neuronal spinal cord segments**.

Segment	Percentage of spinal cord	Cumulative percentage of spinal cord
C1	1.6	1.6
C2	2.2	3.9
C3	3.5	7.3
C4	3.5	10.8
C5	3.5	14.3
C6	3.3	17.6
C7	3.2	20.8
C8	3.4	24.1
T1	3.6	27.7
T2	3.9	31.6
T3	4.4	36
T4	5	41
T5	5.1	46.1
T6	5.6	51.8
T7	5.6	57.4
T8	5.4	62.7
T9	5.1	67.8
T10	4.7	72.4
T11	4.3	76.7
T12	3.9	80.6
L1	3.6	84.2
L2	2.8	87
L3	2.4	89.4
L4	2.2	91.6
L5	1.7	93.3
S1	1.5	94.9
S2	1.6	96.4
S3	1.4	97.8
S4	1.3	99.1
S5	0.9	100

*With data from the articles in Table 2, we calculated the relative proportions of each spinal cord neuronal segment relative to the length of the whole spinal cord*.

*C, cervical; T, thoracic; L, lumbar, S, sacral*.

#### Relative Length of Vertebral Bony Segments

The vertebral bony segments became longer in the caudal direction, with the lumbar vertebrae being the longest. The lumbar vertebrae constitute almost 6% each of the whole vertebral column, or 3.5 cm per segment. The absolute measurement is naturally highly dependent on the length of torso of the individual. The calculated relative length of each vertebral bony segment is presented in Table 4.

**Table 4:** **Relative lengths of human vertebral bony segments**.

Segment	Percentage of vertebral column	Cumulative percentage of vertebral column
C1	3.2^a^	3.2
C2	3.2^a^	6.4
C3	2.8	9.1
C4	2.7	11.9
C5	2.7	14.6
C6	2.6	17.2
C7	3.1	20.2
T1	3.4	23.6
T2	3.7	27.3
T3	3.7	31.1
T4	3.9	34.9
T5	3.9	38.8
T6	4.2	42.9
T7	4.3	47.3
T8	4.5	51.7
T9	4.6	56.3
T10	4.8	61.2
T11	5.1	66.2
T12	5.4	71.7
L1	5.7	77.3
L2	5.8	83.1
L3	5.7	88.8
L4	5.7	94.6
L5	5.5	100

*^a^Approximated*.

*With data from Panjabi et al. (7–9), we calculated the relative proportions of each vertebral bony segment relative to the length of the whole vertebral column. One vertebral bony segment was defined as the vertebra and half of both adjacent intervertebral disks. There was no data for segments C1 and C2, and their respective percentages were approximated using the relative positions of C3 and C4 spinal cord neuronal segments and C3 vertebral bony segment from Cadotte et al. (10), with the assumption that the upper end of the C1 spinal cord neuronal segment was aligned with the upper end of the C1 vertebral bony segment and the termination of the spinal cord was located between vertebral bony segments L1 and L2*.

*C, cervical; T, thoracic; L, lumbar*.

### Relative Positioning of Segments

#### Effect of Corrected Positioning of Transverse Diameter Measurements of the Human Cervical Spinal Cord—*R*-Squared and Bootstrap

Figure 1 shows the alignment of spinal cord neuronal segments and vertebral bony segments calculated by our method. Figure 2A shows a raw positioning using only segment index, and Figure 2B shows our best effort to position measurements of the transverse diameter of the human cervical spinal cord correctly along a normalized craniocaudal axis.

To estimate the effect of the adjustment of craniocaudal position of measurements, we set up two second-degree polynomial regression models. The *R*-squared value for Model 1 (uncorrected positioning) was 68.8% and 86.4% for Model 2 (corrected positioning), which was applied to the corrected data shown in Figure 2B. The 95% confidence intervals for the *R*-squared values were non-overlapping, indicating a robust difference.

The numerical results from the models and bootstraps are presented with the underlying data in Figures 2A,B.

### Weighted Averages

#### Weighted Averages along the Spinal Cord

To combine the cross-sectional measurements and SDs of the human spinal cord from all studies into single estimates, we calculated weighted averages. Figure 5 shows the raw weighted averages and SDs along the spinal cord.

### Population Estimates

#### Continuous Population Estimates along the Spinal Cord

To construct continuous population estimates and achieve further smoothing, a generalized additive model was fit to the weighted averages and weighted SDs.

**Figure 3:**
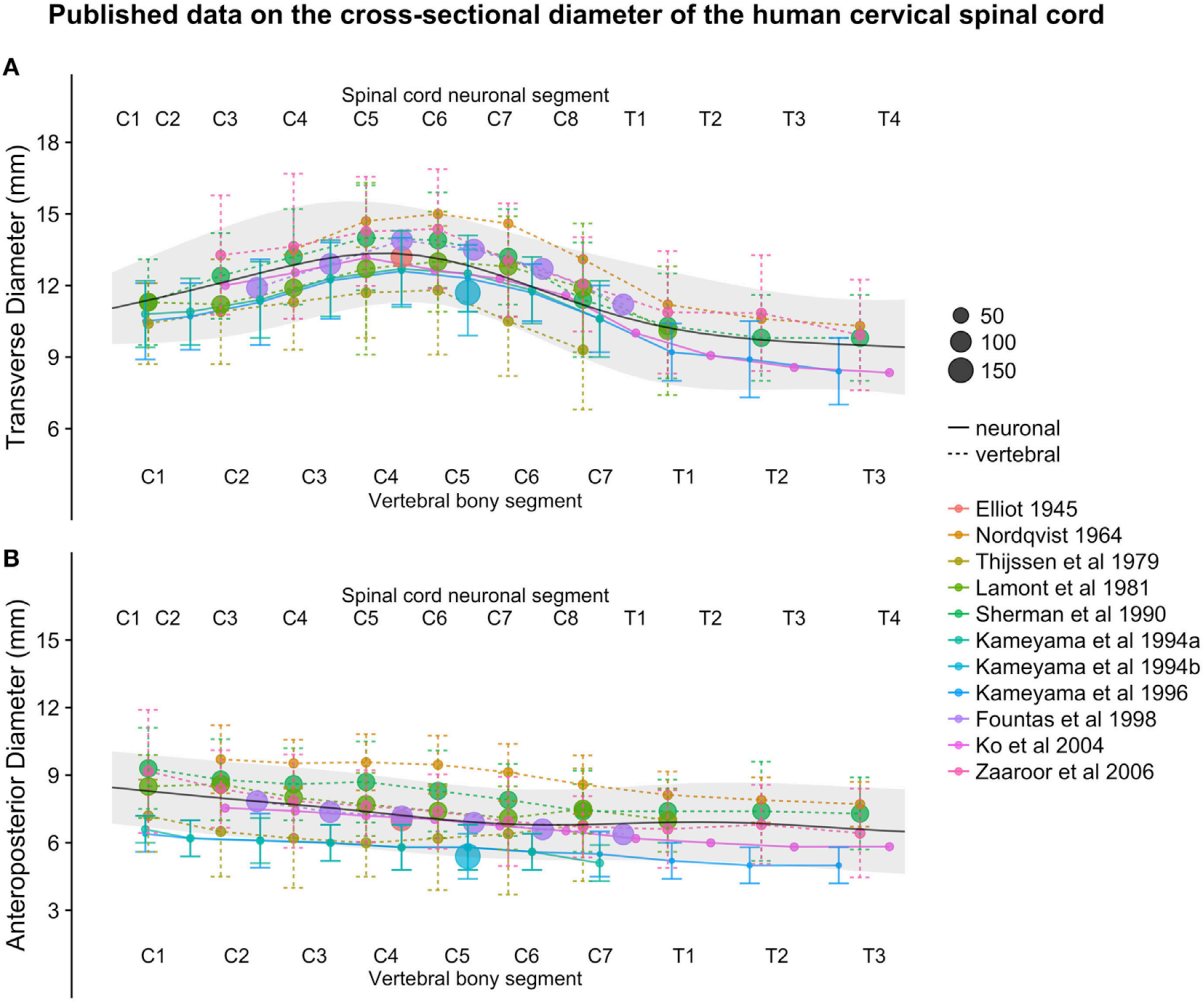
**(A,B)**: figure illustrates measurements of the human cervical spinal cord transverse [panel **(A)**] and anteroposterior diameter [panel **(B)**] from different published studies. The size of the dots represents the number of subjects included in each study. The full black line shows the continuous population estimate from the general additive model, and the gray ribbon represents the population estimate ± 2 standard deviations (SDs) (based on the SDs of the studies).

**Figure 4:**
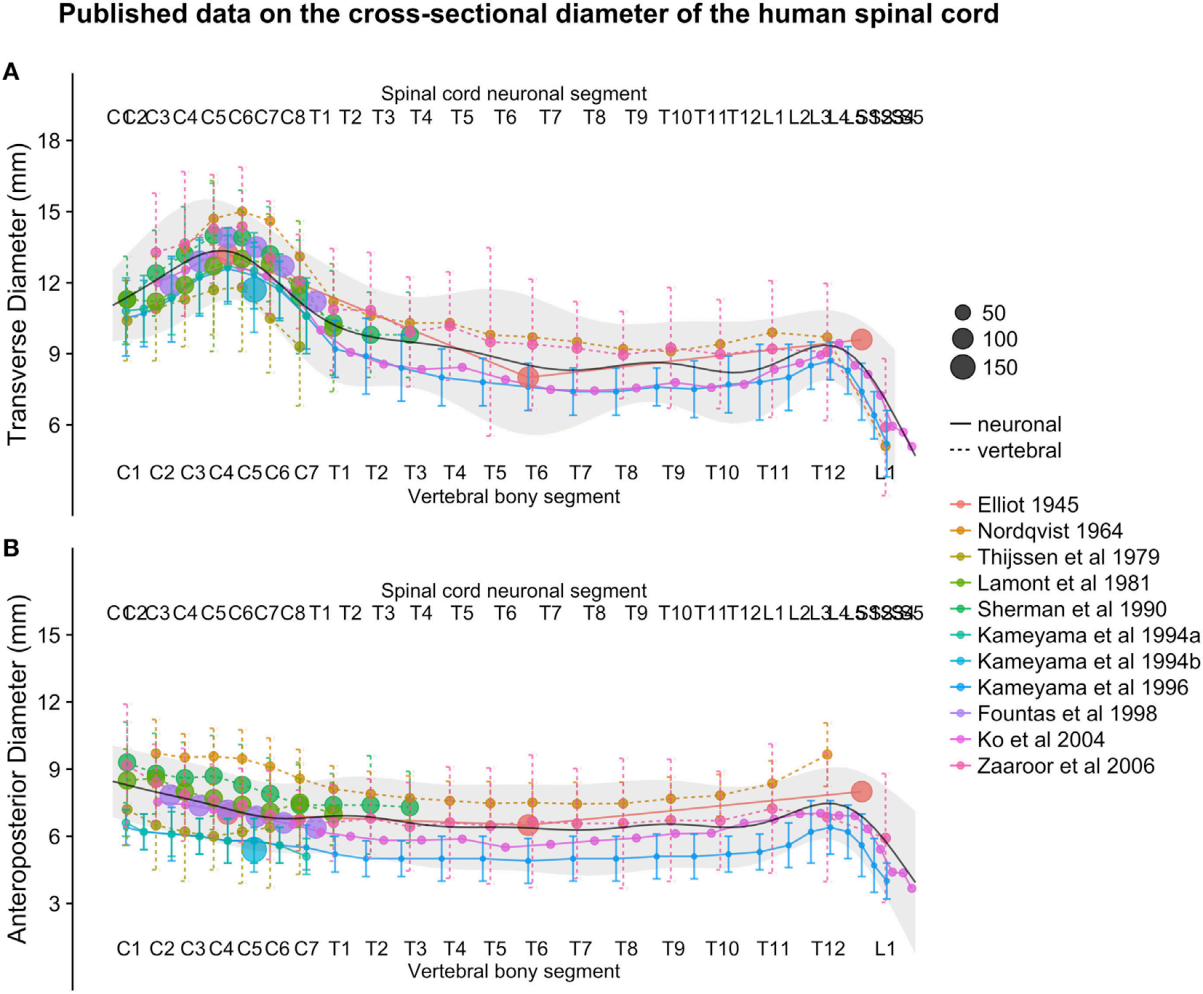
**(A,B)**: figure illustrates measurements of the human spinal cord transverse [panel **(A)**] and anteroposterior diameter [panel **(B)**] from different published studies. The size of the dots represents the number of subjects included in each study. The full black line shows the continuous population estimate from the general additive model, and the gray ribbon represents the population estimate ± 2 standard deviations (SDs) (based on the SDs of the studies).

**Figure 5:**
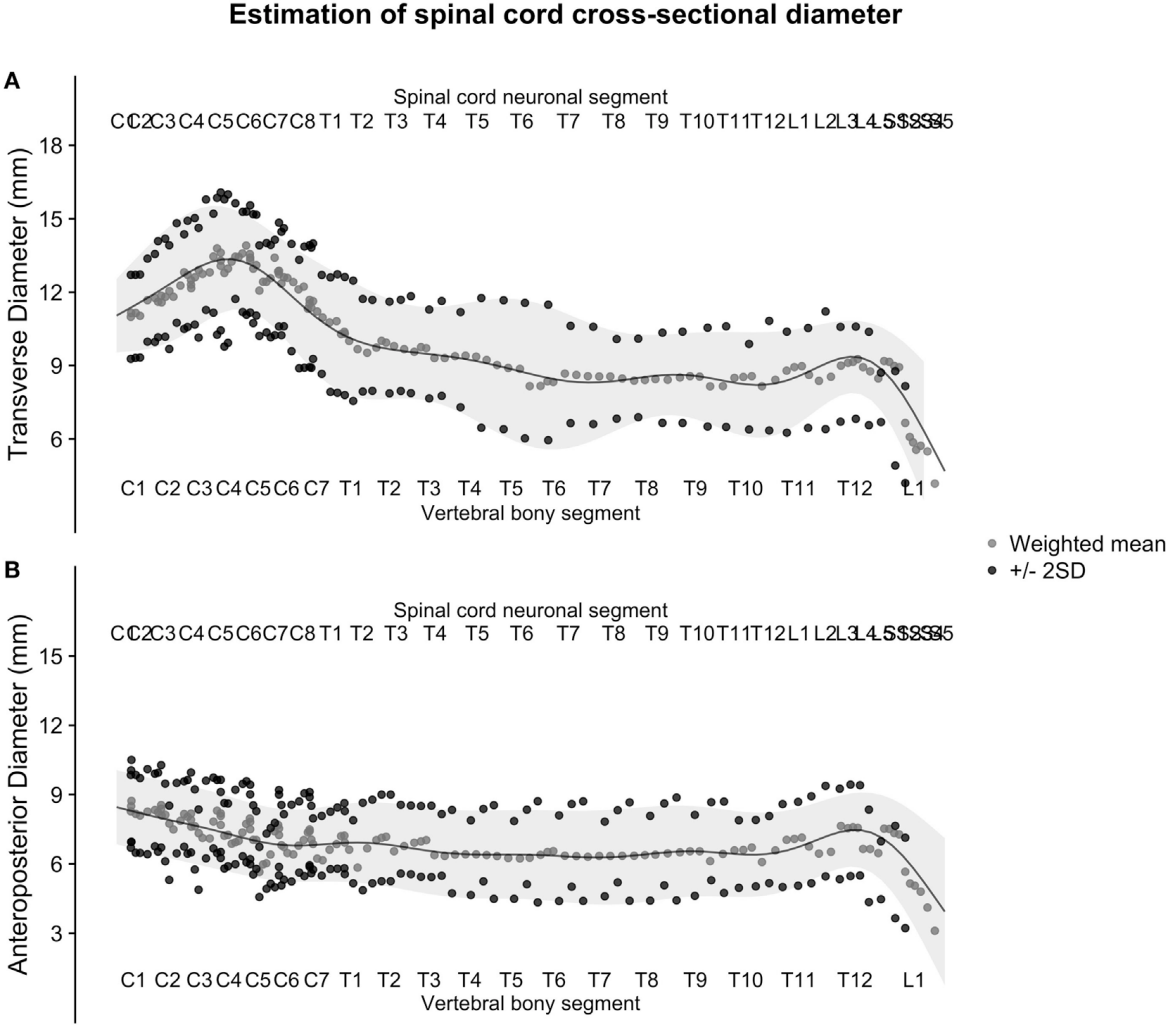
**(A,B)**: figure illustrates the weighted averages of the human spinal cord transverse [panel **(A)**] and anteroposterior diameter [panel **(B)**] from different published studies. The full black line shows the continuous population estimate from the general additive model, and the gray ribbon shows two standard deviations (SDs) from the population estimate based on the SDs of the studies.

The smoothed continuous population estimates of human spinal cord transverse and anteroposterior diameters are shown in Figure 3 (cervical spinal cord with original data from the studies), Figure 4 (whole spinal cord with original data from the studies), and Figure 5 (whole spinal cord with weighted averages and SDs). The transverse diameter of the spinal cord showed the expected shape with a marked cervical intumescence and a smaller lumbar intumescence. The anteroposterior diameter decreased throughout the spinal cord.

#### Point Values of Continuous Population Estimates along the Spinal Cord

To facilitate comparison between our continuous population estimates and other studies, we extracted exact values for each spinal cord neuronal segment as well as vertebral bony segment.

Results for each spinal cord neuronal segment are presented in Table 5 and for vertebral bony segment in Table 6.

**Table 5:** **Estimated spinal cord diameters—spinal cord neuronal segment reference**.

Spinal cord segment	Transverse diameter	Anteroposterior diameter	Number of subjects
C1	11.3 ± 1.7	8.3 ± 1.6	26
C2	11.5 ± 1.9	8.2 ± 1.6	181
C3	12 ± 2.3	8 ± 1.6	318
C4	12.8 ± 2.4	7.7 ± 1.7	362
C5	13.3 ± 2.2	7.4 ± 1.6	234
C6	13.1 ± 1.9	7 ± 1.6	438
C7	12.5 ± 1.9	6.9 ± 1.6	488
C8	11.3 ± 2.2	6.8 ± 1.6	336
T1	10.7 ± 2.3	6.9 ± 1.6	316
T2	10 ± 2.3	6.9 ± 1.7	27
T3	9.6 ± 2	6.8 ± 1.8	131
T4	9.5 ± 1.9	6.6 ± 1.9	131
T5	9.2 ± 2.4	6.4 ± 1.9	65
T6	8.7 ± 3	6.4 ± 1.9	65
T7	8.4 ± 2.7	6.3 ± 2	167
T8	8.3 ± 2.1	6.3 ± 2	77
T9	8.6 ± 1.7	6.5 ± 2	65
T10	8.6 ± 1.8	6.5 ± 2	65
T11	8.3 ± 2.1	6.4 ± 1.9	65
T12	8.2 ± 2.1	6.4 ± 1.8	27
L1	8.6 ± 1.9	6.7 ± 1.7	65
L2	9.1 ± 1.6	7.2 ± 1.6	27
L3	9.4 ± 1.5	7.5 ± 1.6	77
L4	9.3 ± 1.5	7.5 ± 1.6	27
L5	8.8 ± 1.7	7.1 ± 1.8	27
S1	8.4 ± 1.9	6.8 ± 2	129
S2	7.1 ± 2.5	5.8 ± 2.4	65
S3	6.3 ± 2.8	5.2 ± 2.7	27
S4	5.5 ± 3.2	4.6 ± 2.9	15
S5	4.7 ± 3.5	3.9 ± 3.2	15

*Point values of population estimates shown in Figures 3 and 4 are depicted here in numerical format at the craniocaudal position of the middle of each spinal cord neuronal segment. “Number of subjects” indicates the number of measurements found within the craniocaudal limits of each segment and should not be interpreted as an exact “n” for statistical calculations, but rather as an approximation of the amount of data available along the spinal cord*.

*C, cervical; T, thoracic; L, lumbar; S, sacral*.

**Table 6:** **Estimated spinal cord diameters—vertebral column bony segment reference**.

Vertebral column segment	Transverse diameter	Anteroposterior diameter	Number of subjects
C1	11.5 ± 1.9	8.2 ± 1.6	207
C2	12.3 ± 2.4	7.9 ± 1.6	318
C3	13.1 ± 2.4	7.6 ± 1.7	336
C4	13.3 ± 2.1	7.3 ± 1.6	438
C5	13.1 ± 1.9	7 ± 1.6	488
C6	12.1 ± 2	6.8 ± 1.6	336
C7	11 ± 2.3	6.8 ± 1.6	351
T1	10.2 ± 2.4	6.9 ± 1.7	200
T2	9.7 ± 2	6.8 ± 1.8	131
T3	9.5 ± 1.9	6.6 ± 1.9	131
T4	9.2 ± 2.4	6.4 ± 1.9	65
T5	8.8 ± 2.9	6.4 ± 1.9	65
T6	8.4 ± 2.9	6.3 ± 2	167
T7	8.3 ± 2.3	6.3 ± 2	65
T8	8.6 ± 1.7	6.4 ± 2	65
T9	8.6 ± 1.8	6.5 ± 2	77
T10	8.2 ± 2.1	6.4 ± 1.8	80
T11	8.6 ± 1.9	6.7 ± 1.7	92
T12	9.4 ± 1.5	7.5 ± 1.6	119
L1	7.1 ± 2.5	5.8 ± 2.4	251

*Point values of population estimates shown in Figures 3 and 4 are shown here in numerical format at the craniocaudal position of the middle of each vertebral bony segment. “Number of subjects” indicates the number of measurements found within the craniocaudal limits of each segment and should not be interpreted as an exact “n” for statistical calculations, but rather as an approximation of the amount of data available along the spinal cord*.

*C, cervical; T, thoracic; L, lumbar; S, sacral*.

#### Number of Measurements and Relative Contribution of Studies along the Spinal Cord

**Figure 6:**
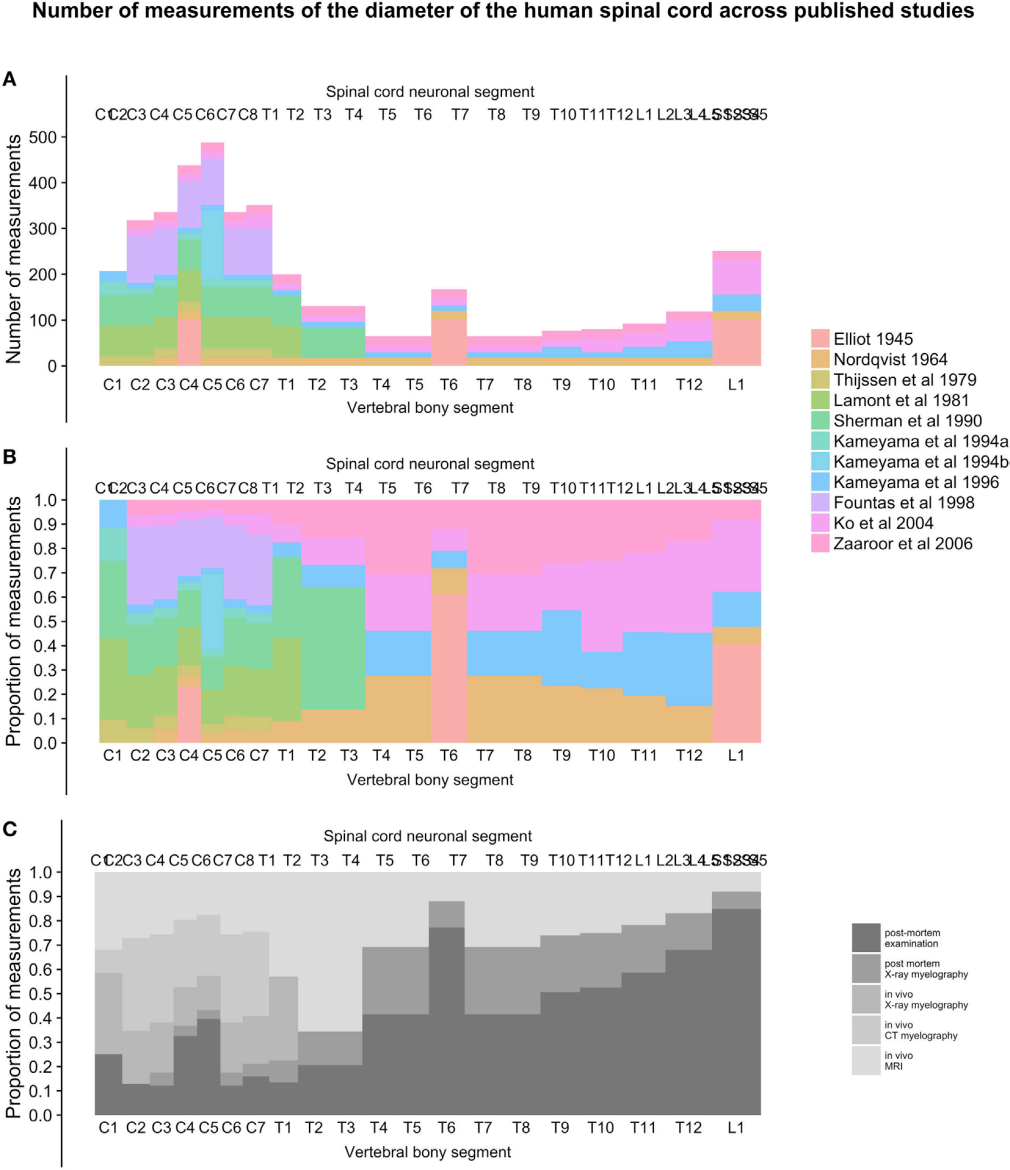
**(A–C)**: figure demonstrates the total number of individual measurements contributed from each study at different points along the craniocaudal axis [panel **(A)**] and the proportional contribution of studies [panel **(B)**] and methods [panel **(C)**] used to measure the diameters of the human spinal cord.

As seen in Figure 6A, the number of measurements in the cervical spinal cord is much greater than in the thoracic, lumbar, and sacral parts, with around 10 times the sample sizes. The proportion of *in vivo* methods is also greater in the cervical spinal cord (Figures 6B,C).

## Discussion

We estimated normal human spinal cord transverse and anteroposterior diameter from previously published data. To compare and combine these different studies, we created and analyzed a conversion method to place measurements correctly along a standardized craniocaudal axis. We created weighted averages of measurements and combined them with a generalized additive model to create a final continuous population estimate of transverse and anteroposterior diameter, as well as the associated SDs along the craniocaudal axis of the entire human spinal cord.

### Studies and Data

#### Studies Included in the Analysis

We included a variety of studies from different eras of research using different methodologies. We deemed this necessary because of the small number of studies available overall and the incomplete coverage of the spinal cord in these studies.

#### Quality of Original Data Included in the Analysis

The reliability of the estimated segmental spinal cord diameters presented is based on the quality of the reported data in the studies included. These reported data were based on either radiology or postmortem examination of the healthy human spinal cord. When implementing a radiological approach for segmental measurements, the delimitation of the cord is vital in order to achieve accurate measurements (18). Lamont et al. found it challenging to delimit the nerve root from the actual spinal cord, which prompted them to measure the whole width of both the cord and the root and to conduct the measurements at the mid-vertebral level only (18). Single reference points imply error consistency throughout the spinal cord but are likely to reduce the quality of the estimate and aggravate the comparison between previously published data. Sherman et al. used a more rigorous approach in obtaining between 10 and 110 samples from each cord level (4). Techniques such as computed myelography allow sectioning down to 13 mm thickness, which is significantly thicker than what is achievable through postmortem studies (17). However, both Thijssen et al. and Sherman et al. emphasized the need for axial radiological sectioning perpendicular to the cord to avoid elongation of the sections (4, 17). Other elements which might influence the quality of radiological measurement are: window settings, concentration of contrast media like computed tomographic myelography (20, 25), and window level and pulse sequence for MRI (20, 26). Finally, cranial parts of the cervical spinal cord are especially difficult to measure using the radiological approach, as overlap with the base of the skull, incisor teeth, and maxilla greatly obstructs vision (18).

Despite the potential for differences in quality between studies, we did not weigh the different studies based on their perceived quality.

### Relative Lengths of Segments

#### Relative Length of Spinal Cord Neuronal Segments

Delimitation of the segments is vital when calculating the length of spinal cord neuronal segments. Donaldson and Davis measured the distance between the uppermost fila of successive nerves in four subjects on the dorsal and ventral aspect of the cord (24). However, Ko et al. measured the distance between the lowermost filament of the just proximal segment and the lowermost filament within each root, based on a sample consisting of 13 males and 2 females (22).

### Relative Length of Vertebral Bony Segments

We have included three studies from the same research group reporting data on vertebral length (7–9). The sample size included in the respective publications was 15 or lower, and additional studies and/or more subjects would have been an advantage. When estimating the height of the vertebral body, the points of measurement are important. Panjabi et al. measured the posterior vertebral body height of cervical vertebras in the midsagittal plane (7). The authors reported that this resulted in an average underestimation of the height of each vertebral body by approximately 2 mm, when comparing to previously reported data (7, 27, 28). The same research group (8) found that the posterior thoracic vertebral body height was consistently one to 2 mm less than that reported by Berry et al. and Scoles et al. (29, 30) but in line with data reported by Cotterill et al. (31). The same applied for lumbar vertebral body posterior height (27, 29, 30). Since we used relative vertebral size rather absolute measurements, a systematic error in measurement is of minor importance. Panjabi et al. did not report the lengths of the C1 and C2 vertebrae. Therefore, we calculated approximate percentages for these vertebrae by using previously published relative positions of segments in the cervical spinal cord (10), termination of the spinal cord between lumbar vertebral bony segments L1 and L2 (11), and the assumption that the upper end of the C1 vertebrae is aligned with the upper end of the C1 neuronal segment. Therefore, the relative proportions of segments C1 and C2 in our model should be interpreted with care.

Because we defined a vertebral bony segment as the vertebra and half of both the adjacent intervertebral disks, our model assumes that intervertebral disks increase in thickness along the craniocaudal axis by same proportion as the vertebrae. This is not an unreasonable assumption, but one that was not backed with any data.

### Relative Positioning of Segments

#### Effect of Corrected Measurement Positioning of the Transverse Diameter of the Human Cervical Spinal Cord—*R*-Squared and Bootstrap

Despite the complexity and shortcomings of our model, with scarce data and reliance on a number of assumptions, the strategy to create a normalized craniocaudal axis for comparison of cross-sectional measurements of the human spinal cord was successful. Success was indicated by the increase in adjusted *R*-squared from 68.8 to 86.4% when comparing the raw positioning using only segment index and our best effort to place measurements based on their calculated position. The increase in adjusted *R*-squared was robust, as shown by the non-overlapping 95% confidence intervals achieved by bootstrapping the adjusted *R*-squared for the two models. The relative positioning of segments along the spinal cord relies heavily on the studies by Cadotte et al. (10) and Boonpirak and Apinhasmit (11).

### Weighted Averages

#### Weighted Averages along the Spinal Cord

The weighted averages were calculated by combining four adjacent measurements. This step was necessary to normalize the number of measurements along the spinal cord before fitting the generalized additive model to decrease problems where sample sizes changed suddenly along the spinal cord.

The number four was reached empirically by the authors and can therefore be questioned. We argue that it combined measurements to a reasonable degree without losing frequency response in the signal. In the measurements of anteroposterior diameter, the small number of measurements resulted in periodical oscillations of the weighted averages in the cervical spinal cord (Figure 5B). This was ameliorated in the next step by fitting the generalized additive model.

#### Weighted SDs along the Spinal Cord

The weighted SDs were calculated by squaring the known SDs to become variances and computing the weighted average variances. Taking the square root of the weighted average variances yielded the weighted SDs. This approach assumes that samples were drawn from the same population, which is probably not entirely true but represented the only practical way of estimating aggregated SDs known to us without the original data.

### Population Estimates

#### Weighted Averages along the Spinal Cord

The continuous population estimates of the transverse and anteroposterior diameter resulting from the combination of the included studies (Figures 3–5) were consistent with the expected shape of the spinal cord (e.g., cervical and lumbar intumescence). The population SDs enclosed almost all data points when plotted as two SDs, giving further confidence that these data were combined with some accuracy.

The choice of parameters for the generalized additive model was reached empirically just like the weighted average. When choosing parameters that accurately described the data, we chose the lowest possible order polynomial that would follow the perceived shape of the spinal cord with some accuracy. This was only evaluated visually and represents a weakness of the approach.

#### Correlation between Spinal Cord Size and Other Morphometrics

It is reasonable to discuss the impact of morphometrics defining body size, such as gender, height and body weight. Sherman et al. confirmed the previously established (32) lack of correlation between body weight, age, and spinal cord size, and that these parameters do not have to be adjusted (4). Kameyama et al. (20) also confirmed that the size of the spinal cord has no correlation with age, height, or body weight by concluding that the relative ratio of the cross-sectional area of each cervical, thoracic, and lumbar segment to that of the C3 are similar between individuals, even with a large interindividual variation in spinal cord size. However, some contradictory results were presented by Kameyama et al. (19), who reported that differences between genders seem to include not only spinal cord length (11) but also the cross-sectional area. They found that the cross-sectional area for C7 was significantly smaller in females when compared to males, hypothesizing that the difference in size of the spinal cord between sexes may be partly explained by the variation in height. However, they could not find any correlation between spinal cord size and body weight.

Individual variation in cord size was substantial between individuals with equal height and resulted in significant positive correlation to cross-sectional area, transverse diameter, and sagittal diameter (19). However, Kameyama et al. found that body weight had no significant correlation to cross-sectional area, diameter, or sagittal diameter. The authors report that age had a slight negative correlation to cross-sectional area and sagittal diameter at C7, but not for transverse diameter at the same level. They hypothesize that age-related degenerative changes may explain the flattening of the cervical spinal cord with age, confirming previously published data (16, 19, 33). We observed that many of the included studies tended to include more males than females, which could have affected our analysis.

In summary, some contradictions seem to exist between the impact of body type characteristics on spinal cord size, but most previous studies have been underpowered to detect all but very strong correlations. Because our present study lacks the raw data, further investigation of predictors for spinal cord size was not possible. An interesting expansion of this study would be to gather all raw data and analyze predictors of size in a larger sample.

### Clinical Implications

#### Clinical Implications of Population Estimates

Continuous population estimates of the transverse and anteroposterior diameters of the spinal cord could be useful in diagnosing and monitoring patients with neurodegenerative and neuroinflammatory diseases. It is known, for example, that patients suffering from multiple sclerosis have a reduced cross-sectional area compared to healthy matched controls (1), but these studies have low power. Without population estimates, it can be difficult to determine whether a specific patient should be considered to have a pathologically small or large spinal cord.

#### Clinical Implications of Model for Relative Segmental Positions

In the future, the model of spinal cord neuronal segment relation to vertebral bony segment could be used to achieve a better understanding of visible localized pathology on MRI in the spinal cord in situations where identification of spinal cord neuronal segments is challenging. This would require a validating study in patients in whom a well-defined pathology of the spinal cord is present and can be correlated to a segmental symptom such as the motor or sensory level of a patient with a spinal cord injury. Such a study is currently being planned in our research group.

#### Clinical Research Implications

Multiple experimental studies for treatment of acute and chronic human spinal cord injuries are in different phases of development (2). In all studies where a premade device, instrumentation, or otherwise physical object needs to be applied to the spinal cord, the population estimates are of importance because they represent the variation in physical dimensions that will be encountered in patients.

Our research group is involved in a clinical trial exploring surgical repair of the human spinal cord (http://ClinicalTrials.gov Identifier: NCT02490501). During the design of the biodegradable device used in the study, knowing population estimates of the human spinal cord was a necessity, and, therefore, we believe that this work can be useful for other groups in similar projects.

## Conclusion

We conclude that segmental transverse and anteroposterior diameters of the healthy human spinal cord from different published sources can be combined on a normalized craniocaudal axis and yield meaningful population estimates with reasonable sample sizes. These estimates could be useful for the routine management of patients with neurodegenerative diseases as well as for clinical research and experimental applications involving surgical spinal cord repair.

## Ethics Statement

This review was based solely on published papers and their reported data. We did not conduct any radiological or postmortem examinations by ourselves. Hence, this review itself is exempt from ethical approval but relies on the ethics of the included studies, which we have found no reason to question.

## Author Contributions

Substantial contributions to the conception or design of the work (AF); or the acquisition (AF), analysis (AF, RH, ET, PM, and MS), or interpretation (AF, RH, ET, PM, and MS) of data for the work. Drafting the work or revising it critically for important intellectual content (AF, RH, ET, PM, and MS). Final approval of the version to be published (AF, RH, ET, PM, and MS). Agreement to be accountable for all aspects of the work in ensuring that questions related to the accuracy or integrity of any part of the work are appropriately investigated and resolved (AF, RH, ET, PM, and MS).

## Conflict of Interest Statement

The authors declare that the research was conducted in the absence of any commercial or financial relationships that could be construed as a potential conflict of interest.

## Abbreviations

CT, computed tomography; MRI, magnetic resonance imaging; C, cervical; T, thoracic; L, lumbar; S, sacral.
